# Optimising retention success: a research team’s experience of following-up participants recruited to a pilot trial through community pharmacies in England

**DOI:** 10.12688/f1000research.25372.2

**Published:** 2021-02-15

**Authors:** Michelle Watson, Anne van Dongen, Catherine Hewitt, Laura Mandefield, Duncan Stewart, Judith Watson, Jim McCambridge

**Affiliations:** 1 Department of Health Sciences, University of York, York, YO10 5DD, UK

**Keywords:** Pharmacy services and practice, Trials/Randomised controlled trials, Health services research

## Abstract

**Background**: The CHAMP-1 ( Community pharmacy: Highlighting Alcohol use in Medication a Ppointments) pilot trial aimed to explore an intervention discussing alcohol during medication consultations with community pharmacists. It presented various challenges regarding patient retention, as participants were recruited by their pharmacist and followed-up remotely by a trained researcher, who they had not met, two months later.  We discuss our actions and experiences of completing follow-up activities.

**Methods**: Community pharmacists recruited patients aged 18 and over, attending a Medicine Use Review (MUR) or New Medicine Service (NMS) consultation, and drinking alcohol at least twice per week. Pharmacies were randomised to conduct their consultations as usual (control), or to incorporate the Medicines and Alcohol Consultation (MAC) intervention. All participants were followed-up by a researcher after two months to complete data collection via telephone or post. We employed standard follow-up strategies, including a plan to text participants with a reminder in advance of their follow-up.

**Results**: Forty-seven of 51 participants (92%) completed the two month follow-up. Thirty-eight (81%) responses were provided by telephone and nine (19%) by post. Of the 38 follow-up calls completed by telephone, 17 (45%) participants were reached at first attempt; 16 (42%) at second attempt; and five (13%) at the third attempt. We observed a high percentage of data completion across telephone and postal collection methods.  Participants were willing to discuss potentially sensitive issues, such as alcohol consumption, anxiety, and depression, with a researcher who was external to the pharmacy team.

**Conclusions**: The results suggest that patients recruited to a trial by community pharmacists are willing to take part in data collection activities, and remote follow-up can be successfully conducted by researchers. The techniques employed to encourage high levels of retention should be investigated further in a larger study, alongside consideration of optimal strategies to collect data within community pharmacies.

## Introduction

Community pharmacies are a dynamic environment with professionals who are keen to provide care and support for a wide range of healthcare service users. There are around 11,600 community pharmacies in England, and 89% of the population are able to reach such facilities within a 20-minute walk ^1^, allowing pharmacies to be at the core of communities and patient care. Over time, the role of a community pharmacist has expanded beyond the traditional dispensing duties ^2^, including taking on wider public health roles and research activities. Research in this setting is, however, not without its challenges, with time constraints and remuneration ^3^ having been reported as difficulties previously. More widely, difficulties in retaining research participants recruited to trials is a common issue and represents a significant risk to the statistical power and analysis of trial results ^4^, while also potentially introducing bias and reducing generalisability ^5^.

The CHAMP-1 ( Community pharmacy: Highlighting Alcohol use in Medication a Ppointments) pilot cluster trial is part of a programme of work which aims to collaborate with the pharmacy profession and patients, to produce an intervention discussing alcohol within routine medication consultations. The design of the trial was informed by pre-trial studies conducted by the CHAMP-1 research team, including observational and interview work with patients and pharmacists ^6, 7^. The pre-trial work guided development and implementation of the intervention, rather than follow-up procedures. Outcome data collection in the trial provided a challenge as participants were recruited by their community pharmacist but followed-up by a trained researcher who the participant had no prior contact with. We used the standard follow-up procedures of York Trials Unit, though were uncertain how successful they would be in these circumstances. 

Full results of the pilot trial and the experiences of the community pharmacists involved are reported elsewhere ^8, 9^. This paper focuses specifically on our experiences of contacting participants and the techniques used in an attempt to maximise our follow-up rate.

## Methods

The pilot trial’s primary clinical outcomes were the total weekly UK units (8 g of ethanol per unit) of alcohol consumption in the week prior to follow-up, and confidence in medications management. Details of the trial’s methodology are reported elsewhere ^8^.

Twenty-seven community pharmacies in Yorkshire, England expressed an interest to be involved in the pilot trial, of which four were excluded (two had previous CHAMP-1 involvement and two did not respond) and 23 were assessed for eligibility. Of these, two were found to be ineligible (one postponed a telephone call three times, the other was not enthusiastic about the focus of the intervention) and 11 were excluded for varying reasons (three were unable to commit to the training days, three were unable to commit time to the study overall, two did not have approval from their manager or organisation, two did not respond, and one was not accredited to complete Medicine Use Reviews (MURs)). Ten pharmacies were deemed to be eligible and were randomised to conduct their Medicine Use Review (MUR) or New Medicine Service (NMS) consultations as usual (control), or to incorporate the Medicines and Alcohol Consultation (MAC) intervention. Five pharmacies were randomised to the control group, and five were randomised to provide the intervention. Randomisation for the trial was at the level of the community pharmacy (with one practitioner per pharmacy). A separate randomisation sequence investigating the methodological feasibility of sending text messages to participants and their effect on retention was generated using block randomisation stratified by pharmacy.

Participant recruitment was conducted by community pharmacists, and all participants were followed-up with a telephone call from a trained researcher two months after entering the trial and having their consultation with the pharmacist. During the telephone call, the trained researcher collected outcome data using a Case Report Form (follow-up questionnaire). The follow-up questionnaire asked participants details about their health and wellbeing. This included a question about alcohol consumption; a potentially sensitive issue. We compared a short alcohol measure consisting of two questions based on alcohol consumption in the last seven days (“On how many days did you have a drink containing alcohol?” and “How many units of alcohol did you drink on a typical day when you were drinking?”), against a long alcohol measure (seven day recall diary). We also collected data regarding the participant’s confidence in medication management (PROMIS) ^10^, medication adherence (ProMAS) ^11^, health related quality of life (EQ5D-5L) ^12^, anxiety (GAD-7) ^13^, and depression (PHQ-8) ^14^.

The research team considered the potential challenges to successful follow-up, such as the two month time lapse between the consultation and follow-up call, and the willingness of participants to speak about their health and wellbeing to someone they did not know. To facilitate trial follow-up, the study team established various procedures to address these such as: asking participants for more than one telephone number if available (e.g. a landline and mobile telephone number); asking for the participant’s preferred days and times for contact; sending a text message to remind them that a researcher would be contacting them in the near future to conduct their follow-up telephone call; attempting to contact participants three times before sending the follow-up questionnaire ^15^ (the same form as that completed by telephone) by post; calling from one single telephone number to enable participants to recognise the number if they were unable to be contacted at the first attempt; and leaving voicemail messages where possible requesting that participants return the call. Participants who completed follow-up were given a £10 ‘thank you’ gift card. This was explained in the patient information sheet ^15^ provided during recruitment and mentioned early in the conversation during the follow-up telephone call.

## Results

The CHAMP-1 pilot trial recruited 51 participants from 10 pharmacies. Of these, 55% were men and 45% were women ^15^. The mean age of those involved was 66.5 years. Forty-seven (92%) participants were successfully followed up at two months. Thirty-eight (81%) of the 47 responses were provided by telephone and nine (19%) participants completed the follow-up questionnaire after being sent it by post, having not responded to the telephone calls. Four participants did not respond to the telephone calls or return the follow-up questionnaire by post and therefore their outcome data was not collected.

All participants provided at least one telephone number and 34 (67%) participants provided a mobile telephone number, and therefore were possibly more likely to be contactable if they were not at the location of their landline telephone. Forty-eight participants (94%) also consented to receiving a text message that would remind them about the follow-up telephone call; however, of these, 15 (31%) did not provide a mobile number to enable this to occur. Due to technical difficulties, only seven of the text messages were sent as planned.

Of the 38 follow-up calls completed by telephone, 17 (45%) participants were successfully reached at the first attempt of contact by the trained researcher; 16 (42%) at the second attempt; and five (13%) at the third attempt. Having made contact, nine of the participants requested that the follow-up call be arranged for a more convenient time and this was scheduled accordingly. Eight of these calls were completed successfully as arranged, with five participants requesting this at the first attempt at contact, and three asking during the second. The ninth participant arranged their follow-up call after one attempt at contact; however, they did not engage with the re-arranged or subsequent telephone calls. If no contact had been made after three call attempts, the follow-up questionnaire, which was the same as that completed by telephone, was posted to the participant.

We observed a high percentage of data completion using both telephone and postal methods, as shown in Appendix 1. The long alcohol measure was completed best by telephone (89% fully completed by telephone, 33% fully completed by post (95% CI: 13.7% to 80.8%; P=0.0064)); however the PROMIS measure ^10^ had fewer items missing when completed by post (100% fully completed by post, 79% fully completed by telephone (95% CI: -10.5% to 36.3%; P=0.1355)). Comparison data regarding the short and long alcohol recall measures are presented in the pilot trial paper ^8^.

## Discussion/conclusions

The trial involved participants with a range of ages and therefore is broadly representative of the type of patients that use community pharmacies. We were unable to meet our recruitment target due to various factors, some of which were beyond our control, and these have been described previously ^8^. As the study was conducted in only one geographical location, this presents a limitation that may hinder generalisability. Whilst this was a small pilot cluster trial, it describes the initiatives used to encourage a successful follow-up rate in potentially challenging circumstances. The results suggest that patients recruited within community pharmacies are willing to complete further data collection activities which do not involve their pharmacy or pharmacist, and are willing to discuss potentially sensitive issues such as alcohol consumption and mental health. Repeated efforts to make contact were required for over half of participants.

Study retention may have been affected by factors largely or entirely outside of our capacity to influence; such as the participant’s view on pharmacists providing advanced services, their relationship with the pharmacist, the number of medicines being taken, co-morbidities, and whether the participant was visiting their regular pharmacy or not. Several of the pharmacists worked in pharmacies that were next to a doctor’s surgery, and participants may expect to have such discussions with a doctor or practice pharmacist, rather than a community pharmacist. A participant’s experience of their MUR or NMS consultation (with or without the MAC intervention) may also have affected our retention rate, as a higher retention rate may be anticipated with a more positive experience.

Follow-up questionnaire completion rates were good, irrespective of whether data was collected over the telephone or by post. Participants had completed the EQ5D-5L and PROMIS measures as part of the recruitment process, and therefore may have felt more comfortable with such questions during follow-up, having seen them previously. Some of the measures used were completed better by telephone, others by post; and such considerations will be important when planning future research, to encourage thorough data completion.

It is important to ensure that all necessary information is collected whilst completing recruitment procedures, as approximately a third of participants consented to receive a text message reminder about their follow-up telephone call, however did not provide a mobile number for this to be sent to. Study documentation was reviewed by the research team during pharmacy visits, however it was not considered appropriate to retrospectively contact participants for their mobile number if they had provided an alternative telephone number.

Future research is needed with larger samples and longer follow-up periods to examine other potential mechanisms that contribute to successful follow-up of trial participants recruited in this clinical setting. Due to the technical difficulties encountered when investigating the methodological feasibility of sending text messages to participants, and their effect on retention; it would be beneficial for this element of work to be repeated. Future strategies to improve retention may involve considering if and how pharmacists and/or pharmacy staff can be involved in follow-up and retention activities; whether data collection completed in pharmacies can be improved by using electronic resources; and whether questionnaire completion rates can be improved by offering web or app based access to them. 

## Data availability

### Underlying data

Open Science Framework: CHAMP-1 Pilot Retention Data. https://doi.org/10.17605/OSF.IO/9DQ4W ^15^.

This project contains the following underlying data:

- CHAMP-1 Pilot Retention Data.csv
- Participant demographic data.csv
- Appendix 1 - Data completion for follow up questionnaires by method of completion

### Extended data

Open Science Framework: CHAMP-1 Pilot Retention Data. https://doi.org/10.17605/OSF.IO/9DQ4W ^15^.

This project contains the following extended data:

- CHAMP1_FUpLong_v2 (49582 – Activated, VersiForm)_Reference.pdf (follow-up questionnaire)
- CHAMP1_FUpShort_v2 (13283 – Activated, VersiForm)_Reference.pdf (follow-up questionnaire)
- REFERENCE 2A CHAMP-1 Pilot Patient Consent Form Version 2.0 07.05.2019.pdf
- REFERENCE 2A CHAMP-1 Pilot Patient Information Sheet Version 2.0 07.05.2019.pdf

Data are available under the terms of the Creative Commons Zero "No rights reserved" data waiver (CC0 1.0 Public domain dedication).
