# Supplementary material for: Optimising retention success: a research team’s experience of following-up participants recruited to a pilot trial through community pharmacies in England
Source: F1000Res. 2021 Feb 15;9:1042. Originally published 2020 Aug 25. [Version 2] doi: 10.12688/f1000research.25372.2 (PMC7941093; doi:10.12688/f1000research.25372.2)
Supplement: Peer review history

# Peer review: Reviewer response for version 2

**DOI:** 10.5256/f1000research.54527.r79563

**Published:** 2021-03-08

**Authors:** Roberta Littleford

**Affiliations:** 1 The University of Queensland, Brisbane, Australia

I would like to thank the authors for considering the reviewer’s comments to produce a clear report of their embedded sub-study while directing the reader to supplementary information covering the pilot and main studies.

 I support the revised version and have no further comments.

Is the work clearly and accurately presented and does it cite the current literature?

No

If applicable, is the statistical analysis and its interpretation appropriate?

Not applicable

Are all the source data underlying the results available to ensure full reproducibility?

Yes

Is the study design appropriate and is the work technically sound?

Partly

Are the conclusions drawn adequately supported by the results?

Partly

Are sufficient details of methods and analysis provided to allow replication by others?

No

Reviewer Expertise:

Clinical Trial Specialist.

I confirm that I have read this submission and believe that I have an appropriate level of expertise to confirm that it is of an acceptable scientific standard.

# Peer review: Reviewer response for version 2

**DOI:** 10.5256/f1000research.54527.r79562

**Published:** 2021-03-03

**Authors:** Christopher Partlett

**Affiliations:** 1 Nottingham Clinical Trials Unit, Faculty of Medicine and Health Sciences, University of Nottingham, Nottingham, UK

I would like to thank the authors for carefully considering my initial comments and giving such a clear and detailed response.

 I am happy that the revised version addresses my initial comments and I have no further comments to make.

Is the work clearly and accurately presented and does it cite the current literature?

Partly

If applicable, is the statistical analysis and its interpretation appropriate?

Not applicable

Are all the source data underlying the results available to ensure full reproducibility?

Yes

Is the study design appropriate and is the work technically sound?

Yes

Are the conclusions drawn adequately supported by the results?

Yes

Are sufficient details of methods and analysis provided to allow replication by others?

Yes

Reviewer Expertise:

Medical Statistics & Trial Methodology.

I confirm that I have read this submission and believe that I have an appropriate level of expertise to confirm that it is of an acceptable scientific standard.

# Peer review: Reviewer response for version 1

**DOI:** 10.5256/f1000research.27996.r74512

**Published:** 2020-12-18

**Authors:** Roberta Littleford

**Affiliations:** 1 The University of Queensland, Brisbane, Australia

The article’s aim is to present the research team’s experience of following-up participants recruited into a pilot trial assessing an intervention discussing alcohol consumption habits during routine medication consultations within a community pharmacy environment. There are several issues the authors should consider addressing.

 The manuscript’s narrative focuses on the overarching main study, thereby omitting details required to describe the presented articles aims.

 The premise of optimising retention strategies and therefore retention success are not systematically studied or quantified.

 The authors present the important role that community pharmacists had in the main study's intervention, but pharmacists are out with the retention and follow-up scope and the authors do not postulate if intervention delivery had an impact on retention.

 Standard follow-up strategies were employed.

 The abstract will require to be amended if the recommendations provided below are addressed.

**Introduction:** The introduction mainly addresses the challenges related to pharmacists being researchers, but their role is limited within the scope of the retention and follow-up strategies employed, which were conducted by University researchers.

 This section would benefit from a description of the literature that was considered when designing the follow-up strategies, and report upon the outcomes from the pre-trial work that were implemented into the design. 

**Methods: **This section concentrates on the overarching main study, providing significant detail related to sample size, randomisation, and analysis (which can be addressed separately in the main study manuscript), thereby limiting information required to address the retention and follow-up methodology.

 The primary outcome of the main study could be presented to provide some context.

 This section requires to be rewritten to incorporate a robust methods section of the retention and follow-up strategies employed.

 Including a clarification of the randomisation methods employed for SMS, consent and an outline of the actual method(s) used - automated manual by pharmacists or researcher. Further details here would assist in describing the limitations in the discussion.

 The methods section does not currently describe the validation status of the questionnaires used, including self/researcher completion and how the visualisation of the units of alcohol and VAS were addressed during telephone follow-up calls versus in-person completion (the results/discussion section should address any noted differences).

 The tense within the section varies throughout; this section should use past tense.

**Discussion**

 The optimisation and limitations of the project should be addressed in more detail.

 It may be prudent for the authors (with access to the main study results) to consider and discuss elements that may have impacted retention and follow-up; intervention delivery, impact of tokens, number of medicines, co-morbidities, usual pharmacy/new pharmacy, location within-GP practice or independent.

 The authors should consider expanding on possible future strategies for trials, for example including pharmacists as part of the follow-up and retention success strategies, self-completion contact details, web/app questionnaire completion. 

 The rationale of failing to recruit the required sample size (63%) for the main study should be addressed, and also providing the reasons for pharmacy ineligibility and criteria for exclusion, leading to 13/23 (56%) of pharmacies being excluded. These factors may impact future success strategies. 

 There appears to have been significant training employed with weekly follow-up at pharmacies by the research team. However, the manuscript does not report if data monitoring was conducted, which may have identified the 33.3% missing mobile numbers. 

 The authors should consider adding the project timelines to give context to trial efficiency.

Is the work clearly and accurately presented and does it cite the current literature?

No

If applicable, is the statistical analysis and its interpretation appropriate?

Not applicable

Are all the source data underlying the results available to ensure full reproducibility?

Yes

Is the study design appropriate and is the work technically sound?

Partly

Are the conclusions drawn adequately supported by the results?

Partly

Are sufficient details of methods and analysis provided to allow replication by others?

No

Reviewer Expertise:

Clinical Trial Specialist.

I confirm that I have read this submission and believe that I have an appropriate level of expertise to confirm that it is of an acceptable scientific standard, however I have significant reservations, as outlined above.

# Response

**Published:** 2021-02-05

**Authors:** Michelle Watson

**Affiliations:** University of York, UK, UK

The authors of the manuscript thank the peer reviewers for their comments and have made the following changes based on their suggestions:

**Abstract**

- We have updated the abstract to reflect the changes made to the manuscript based on peer review comments.

**Introduction**

- We have added that loss to follow-up presents a risk to generalisability and may introduce bias.
- We have added a statement advising that the pre-trial work guided development and implementation of the intervention, and did not investigate follow-up procedures; therefore standard procedures were followed.  The paper references the pre-trial work publications should readers wish to look into this further.

**Methods**

- We have removed the methodology for the pilot trial and included a reference to the publication describing this instead. 
- We have added the reasons for excluding pharmacies.
- We have added further information about the randomisation methods.
- We have stated the measures which were included in the follow-up questionnaire.
- We added details about the follow-up questionnaire and measures used.

**Results**

- We have presented data regarding telephone or postal completion of the follow-up questionnaire.

**Discussion**

- We have again provided a reference to the pilot trial publication as this covers many of the points raised during review.
- We have reflected further on the factors that may have affected retention and the limitations of our work, including geographical spread, amongst others.
- We have discussed the data regarding telephone or postal completion of the follow-up questionnaire.
- We have discussed potential follow-up and retention strategies that future research could focus on.

# Peer review: Reviewer response for version 1

**DOI:** 10.5256/f1000research.27996.r72701

**Published:** 2020-10-22

**Authors:** Christopher Partlett

**Affiliations:** 1 Nottingham Clinical Trials Unit, Faculty of Medicine and Health Sciences, University of Nottingham, Nottingham, UK

The authors present an interesting case study exploring the feasibility of conducting follow-up for a pilot randomised trial within the community pharmacy setting. While I think this article is certainly worthy of indexing, I have some recommendations to improve the article.

 Firstly, and most importantly, I found that the methods section (of both the abstract and the main article) read similarly to the methods section of a main trial publication. Since the article is focussing on the experience of following-up participants, the methods section should focus on this aspect of the trial. For instance, the final paragraph of the methods section is most pertinent to this article. Other important aspects of trial design would be better placed in the background, while less relevant aspects of the trial design could be omitted completely (and perhaps replaced with a reference to the trial protocol). This change would significantly improve the overall readability of the article.

 I have listed some other minor comments below:

- The authors state in the final paragraph of the introduction that loss to follow-up represents a significant risk to statistical power and analysis. I would add that it also presents a potential risk to the generalisability of the trial findings.
- A limitation of the study is a lack of geographical spread, which may hinder generalisability of the findings within this article. This should be noted in the discussion.
- Ideally, the reasons for exclusion and ineligibility of pharmacies should be listed.

Is the work clearly and accurately presented and does it cite the current literature?

Partly

If applicable, is the statistical analysis and its interpretation appropriate?

Not applicable

Are all the source data underlying the results available to ensure full reproducibility?

Yes

Is the study design appropriate and is the work technically sound?

Yes

Are the conclusions drawn adequately supported by the results?

Yes

Are sufficient details of methods and analysis provided to allow replication by others?

Yes

Reviewer Expertise:

Medical Statistics & Trial Methodology.

I confirm that I have read this submission and believe that I have an appropriate level of expertise to confirm that it is of an acceptable scientific standard, however I have significant reservations, as outlined above.
